# Effectiveness of Treatment Approaches in COVID-19 Pneumonia: A Comparative Evaluation between a Specialized Center and Conventional Hospitals

**DOI:** 10.3390/healthcare12141365

**Published:** 2024-07-09

**Authors:** Rodolfo Romero Pareja, Martín S. Ruiz Grinspan, María Lorena Castro Arias, Raquel García Hernández, Francisco Javier Martín Sánchez, Esther Álvarez-Rodríguez, Virginia Álvarez Rodríguez, Iria Minguens, Ana María Martínez Molina, Rosario Torres Santos-Olmo, Sixto Aranda, Enrique Torres Rodríguez, Carmen Gimeno Galindo, Israel J Thuissard-Vasallo, Javier Marco Martínez

**Affiliations:** 1 Hospital Emergencias Enfermera Isabel Zendal, 28055 Madrid, Spain; javier.marco@salud.madrid.org; 2 Faculty of Biomedical and Health Sciences, Universidad Europea de Madrid, 28670 Madrid, Spain; israeljohn.thuissard@universidadeuropea.es; 3 Hospital del Henares, 28822 Madrid, Spain; martinsebastian.ruiz@salud.madrid.org; 4 Hospital Universitario 12 de Octubre, 28041 Madrid, Spain; lcastroa@salud.madrid.org; 5 Hospital Universitario Puerta de Hierro-Majadahonda, 28222 Madrid, Spain; raquelgh1990@hotmail.com; 6 Hospital Clínico Universitario San Carlos, 28040 Madrid, Spain; fjjms@hotmail.com; 7 Hospital Universitario Severo Ochoa, 28914 Madrid, Spain; esther.alvarez@salud.madrid.org; 8 Hospital Universitario de Getafe, 28905 Madrid, Spain; valvarez@salud.madrid.org; 9 Hospital General Universitario Gregorio Marañón, 28007 Madrid, Spain; iria.miguens@gmail.com; 10 Hospital Universitario de La Princesa, 28006 Madrid, Spain; ammolina@salud.madrid.org; 11 Hospital Universitario La Paz, 28046 Madrid, Spain; rosario.torres@salud.madrid.org; 12 Hospital del Sureste, 28500 Madrid, Spain; sisidro.aranda@salud.madrid.org; 13 Hospital de El Escorial, 28200 Madrid, Spain; enrique.torres@salud.madrid.org; 14 Hospital Universitario Infanta Leonor, 28031 Madrid, Spain; mariacarmen.gimeno@salud.madrid.org

**Keywords:** COVID-19, healthcare management, mechanical ventilation, pneumonia, mortality

## Abstract

Background: The early stages of the COVID-19 pandemic overwhelmed general hospitals in Spain. In response, a dedicated hospital for COVID-19 care, the Hospital de Emergencias Enfermera Isabel Zendal (HEEIZ), was established. This study aimed to compare clinical outcomes of COVID-19 patients treated at the specialized HEEIZ with those at conventional general hospitals (CGHs) in Madrid, Spain. Methods: The study was a prospective, observational cohort study including COVID-19 patients admitted to the HEEIZ and 14 CGHs (December 2020 to August 2021). Patients were assigned based on hospital preference. Clinical data were collected and analyzed using multivariate regression to assess primary and secondary outcomes, including hospital mortality, need of invasive mechanical ventilation (IMV), and pharmacological treatments. Results: The HEEIZ cohort (*n* = 2997) was younger and had lower Charlson comorbidity scores than the CGH cohort (*n* = 1526). Adjusted HEEIZ hospital mortality was not significantly higher compared with CGHs (OR: 1.274; 95% CI: 0.781–2.079; *p* = 0.332). Conclusions: During the study period, patients admitted to the HEEIZ showed no significant differences in clinical outcomes, compared with patients admitted at CGHs. These results might support the use of specialized centers in managing pandemic surges, allowing CGHs to handle other needs.

## 1. Introduction

The coronavirus disease 2019 (COVID-19) pandemic was one of the most significant health crises of our time and generated intense pressure on healthcare systems worldwide [1,2]. Even though effective vaccines were eventually developed [3,4], all affected countries faced considerable challenges in managing the clinical aspects of the disease at each stage of the pandemic [5,6]. In Spain, several measures were implemented to control the impact of COVID-19 in the public health system, including lockdowns, massive testing, contact tracing, and vaccination campaigns [7,8,9]. Despite these efforts, the clinical management of COVID-19 patients remained a significant challenge, necessitating the establishment of dedicated healthcare facilities [10].

In the early stages of the pandemic, conventional general hospitals (CGHs), overwhelmed with patients, faced significant challenges [11]. Many lacked the necessary medical equipment and supplies to manage critically ill patients. The supply of personal protective equipment and the insufficient capacities of intensive care units (ICUs) and respiratory intermediate care units (RICUs) were, in particular, of paramount concern [12]. Specialized COVID-19 units were created in CGHs across the country, and dedicated teams of healthcare professionals were trained to care for these patients, each center having its own protocol in the management and treatment of COVID-19 patients. However, this had some negative consequences on other hospital units, causing non-COVID-19 patients to face longer waiting times when receiving treatments and surgeries [13]. To mitigate this burden, several hospitals were specifically designed for the treatment of COVID-19 patients, allowing CGHs to focus on other medical conditions and emergencies. Some countries, like China and Spain, opened dedicated hospitals for the care of COVID-19-infected patients. Other sites, such as hotels, were also adapted to allow potential contacts to be isolated [14]. One of these dedicated centers was the Hospital de Emergencias Enfermera Isabel Zendal (HEEIZ) in the Autonomous Region of Madrid, a public emergency center set up in December 2020 to handle the urgent demands of the pandemic. The HEEIZ is one of the largest hospitals in Europe dedicated solely to COVID-19 management, staffed by specialists in various medical fields, such as pulmonology, infectious diseases, and intensive care, in order to ensure that patients receive high-quality care [15]. It utilizes single, standardized protocols, ensuring homogenous management across all patients [15].

This study aims to fill the gap in understanding the effectiveness of dedicated COVID-19 treatment centers compared to conventional hospitals. Specifically, clinical management measures for COVID-19 patients transferred and admitted to the HEEIZ were compared with measures taken for those who refused HEEIZ transfer and were admitted to 14 reference CGHs in the same region, covering a healthcare area of 3,580,476 inhabitants, representing over half (53%) of the total population of the Autonomous Region of Madrid (6,750,336 people). A comprehensive set of clinical and demographic variables was analyzed and compared between these two healthcare settings with the objectives of providing insights into the benefits and limitations of specialized healthcare facilities during pandemics, and guiding future healthcare policy and management strategies in similar circumstances.

## 2. Methods

### 2.1. Study Design

This was a descriptive, prospective, observational cross-sectional cohort study conducted on COVID-19 patients admitted to the HEEIZ and 14 other hospitals of the public network of the Autonomous Region of Madrid between 11 December 2020 and 31 August 2021. The objective was to compare the clinical management and the outcomes of patients treated in a specialized COVID-19 center versus the clinical management and outcomes for those treated in CGHs.

### 2.2. Study Setting

The HEEIZ, a dedicated infectious diseases treatment hospital, has a maximum capacity of 1000 beds, including an ICU and an RICU. The other 14 CGHs are standard hospitals with emergency departments, located in all health areas of the Autonomous Region of Madrid. COVID-19 clinical management protocols varied widely among these centers, despite available guidelines intended to unify criteria [16].

### 2.3. Study Patients

The following inclusion criteria were used: (1) patients ≥18 years of age; (2) SARS-CoV-2 infection confirmed by PCR; (3) requirement for hospitalization for pneumonia or respiratory failure; (4) patient with hemodynamic and respiratory stability; (5) previous history of comorbidities currently controlled. The exclusion criteria were: (1) functional or cognitive impairment; (2) pregnancy; (3) immunodepression; (4) Glasgow Coma Scale < 15 points; or (5) active oncological disease. Patients were initially seen in the emergency departments of CGHs and, if eligible, offered admittance to the HEEIZ by their treating physician.

### 2.4. Study Procedures

Patients meeting the inclusion criteria were proposed for transfer to the HEEIZ. Patients who gave their consent for the transfer were assigned to the HEEIZ group, and those who declined, to the CGHs group. On admission, patients underwent a thorough clinical assessment and were managed according to the updated protocols of their respective hospitals. The HEEIZ admission criteria ensured that patients had stable comorbidities and low risk of ICU admission, which helped to minimize transfers back to CGHs. Patients were followed-up with until hospital discharge. The clinical management protocol at the HEEIZ was updated periodically based on the latest scientific evidence and recommendations from health organizations such as the World Health Organization (WHO), the European Centre for Disease Prevention and Control (ECDC), and the Spanish Ministry of Health [17,18,19].

### 2.5. Study Measures

The primary outcome measure was the comparison of mortality rates between the two cohorts. Secondary outcomes included the need for invasive mechanical ventilation (IMV); IMV–death, a composite outcome of poor progress (need for IMV, or hospital mortality); use of pharmacological treatments for COVID-19 during hospital stay (including antivirals, immunosuppressants, antibiotics, anticoagulants, and any regimen or type of corticosteroid therapy); clinical complications (e.g., respiratory failure, sepsis, heart failure, acute coronary syndrome, and renal failure; see criteria and thresholds in Supplementary Table S1); and length of hospital stay.

### 2.6. Covariates

Covariates included demographic characteristics (age, sex, and estimated weight), comorbidities [Charlson index [20], obesity, and chronic obstructive pulmonary disease (COPD)]; clinical parameters at admission (systolic and diastolic blood pressure, heart rate, baseline oxygen saturation, respiratory rate, and body temperature); laboratory parameters [C-reactive protein (CRP), glutamyl oxaloacetic transaminase (GOT), glutamyl pyruvic transaminase (GPT), D-dimer, creatinine, sodium, ferritin, lactate dehydrogenase (LDH), and leukocytes and lymphocytes]; need for advanced respiratory support [high flow oxygen nasal cannula, noninvasive positive pressure ventilation (NPPV), or invasive mechanical ventilation], destination at hospital discharge (home, social health center, or hospital transfer); number of referrals in the last two months; number of COVID-19 related referrals; admission after referral; and number of days with symptoms prior to admission.

### 2.7. Statistical Analysis

We followed the Strengthening the Reporting of Observational Studies in Epidemiology (STROBE) statement guidelines for observational cohort studies [21]. Since this was an observational study conducted in the context of a challenging health crisis, the research team opted to include the largest possible number of patients without a formal sample size calculation. Quantitative variables are expressed as mean ± standard deviation (SD) or median [25–75% interquartile range (IQR)], based on the Kolmogorov-Smirnov normality analysis, and qualitative variables as absolute (*n*) and relative (%) frequencies. For the comparative analysis between groups of qualitative variables, the chi-squared test was used. For the comparison of quantitative variables, the Student’s T or Mann–Whitney U test were used, depending on the parametric behavior.

Kaplan–Meier overall survival curves until day 30 were computed and compared using log-rank tests. A multivariate regression analysis was performed to identify which variables contributed significantly to the differences in mortality between cohorts, and adjusted by the following baseline characteristics at hospital admission: age, sex, Charlson index, obesity, and number of days with symptoms prior to admission. Statistical analyses were performed using IBM SPSS software v.27 (SPSS Inc., Chicago, IL, USA). All analyses were two-tailed, and statistical significance was determined by whether the confidence interval (CI) excluded 1.

### 2.8. Ethical and Legal Aspects

This study was designed in line with the Declaration of Helsinki and current Spanish regulations for the protection of digital rights and the general regulation of data protection. Standards of Good Clinical Practice and current legal regulations were followed. The local Ethics Committee approved the study (Code: C.P.MIR/HEEIZ_2021_02-C.I. 21/023-E). Since this is a non-interventional study that used de-identified patient data, the Committee decided that obtaining informed consent from patients/representatives was not required.

## 3. Results

### 3.1. Number of Hospital Admissions

In 2021, the Autonomous Region of Madrid experienced three pandemic waves (January–February, April, and July–August) during which the HEEIZ provided RICUs and COVID-19 specific care for 529, 581, and 378 patients at the respective peaks of occupancy (Supplementary Figure S1A). The percentage of hospital admissions to the HEEIZ, compared to those for the entire Autonomous Region of Madrid [22], grew progressively in each successive wave of 2021 (Supplementary Figure S1B), with over 15%, 25%, and 35% of new COVID-19 admissions going to the HEEIZ in the first, second, and third wave, respectively.

### 3.2. Patient Demographics

The study population consisted of 4523 patients, with 2997 in the HEEIZ cohort and 1526 in the CGH cohort (Supplementary Figure S2). The demographic characteristics of the patients included in this study are shown in Table 1. The overall population of patients in both cohorts was mainly composed of men (58.4%). The mean age of patients admitted to the HEEIZ was significantly lower compared than that of CGHs (median ages of 52 (20) and 59 (19) years, respectively, *p* < 0.001). No significant differences were observed in the percentage of the sexes between the two cohorts.

### 3.3. Baseline Clinical Characteristics

Baseline clinical characteristics of the patients are presented in Table 1. The mean Charlson index, which measures comorbidities, was significantly lower in the HEEIZ cohort (0.5 ± 1) compared to the CGH cohort (1 ± 1.5) (*p* < 0.001). The prevalence of obesity was significantly lower in the HEEIZ cohort (14.8% vs. 20.3%, *p* < 0.001), whereas the prevalence of COPD was higher (10.9% vs. 8.7%, *p* = 0.018). Patients in the HEEIZ cohort had a slightly longer duration of symptoms before admission compared to the CGH cohort (median 8 (6–10) vs. 7 (5–10) days, *p* < 0.001). On admission, HEEIZ patients had lower systolic blood pressure (124 mmHg vs. 129.5 mmHg, *p* < 0.001) and heart rate (89 BPM vs. 92 BPM, *p* < 0.001) compared to CGH patients. Baseline oxygen saturation was also slightly higher in the HEEIZ cohort (95% vs. 95%, *p* < 0.001).

### 3.4. Comparison of Administered Treatments and Complications

**Figure 1:**
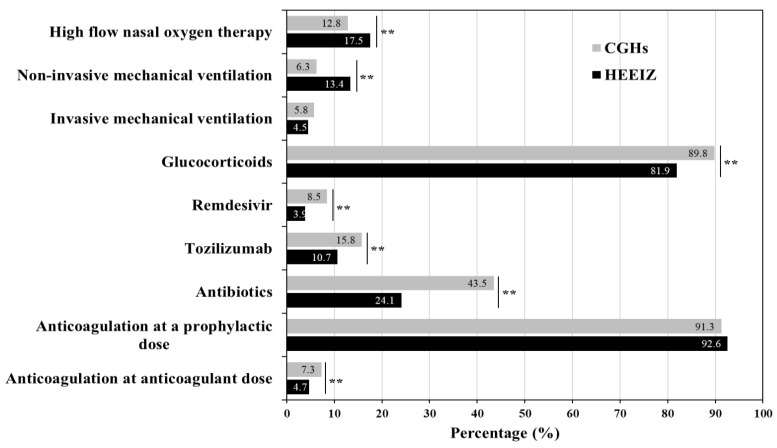
Comparison of treatments administered during hospitalization. **: *p* < 0.001; CGHs: conventional general hospitals; HEEIZ: Hospital de Emergencias Enfermera Isabel Zendal.

Several differences were observed in the clinical management of the two cohorts (Figure 1). Patients at the HEEIZ received high flow nasal oxygen therapy and nonIMV more frequently, compared to patients at CGHs (*p* < 0.001). However, there was no difference in the percentage of patients receiving IMV. In contrast, patients at the HEEIZ less frequently received glucocorticoids, antibiotics, antivirals, and anti-inflammatory drugs, compared to those at CGHs (*p* < 0.001). Over 90% of patients in both populations received prophylactic anticoagulants. The percentage of patients receiving anticoagulants at anticoagulant doses was higher in CGHs compared to the HEEIZ (*p* < 0.001).

A significantly lower percentage of patients at the HEEIZ presented respiratory failure, sepsis, or renal failure, compared to those at CGHs (*p* < 0.001), but there was no statistical difference in the rates of other complications between the two cohorts. The number of referrals was higher in CGHs (*p* < 0.001), including the percentage of COVID-19-related referrals (*p* = 0.018). 

### 3.5. Clinical Outcomes

Crude hospital mortality was significantly lower in the HEEIZ compared with the CGHs (1.5% versus 3.3%, respectively, *p* < 0.001). The composite outcome IMV–death was also significantly lower at the HEEIZ compared to CGHs (4.9% versus 7.6%, respectively; *p* < 0.001). The median HEEIZ hospitalization time was one day shorter than for CGHs (Table 2). Among patients discharged alive, a higher percentage of patients were discharged to their homes (*p* < 0.001). 

**Figure 2:**
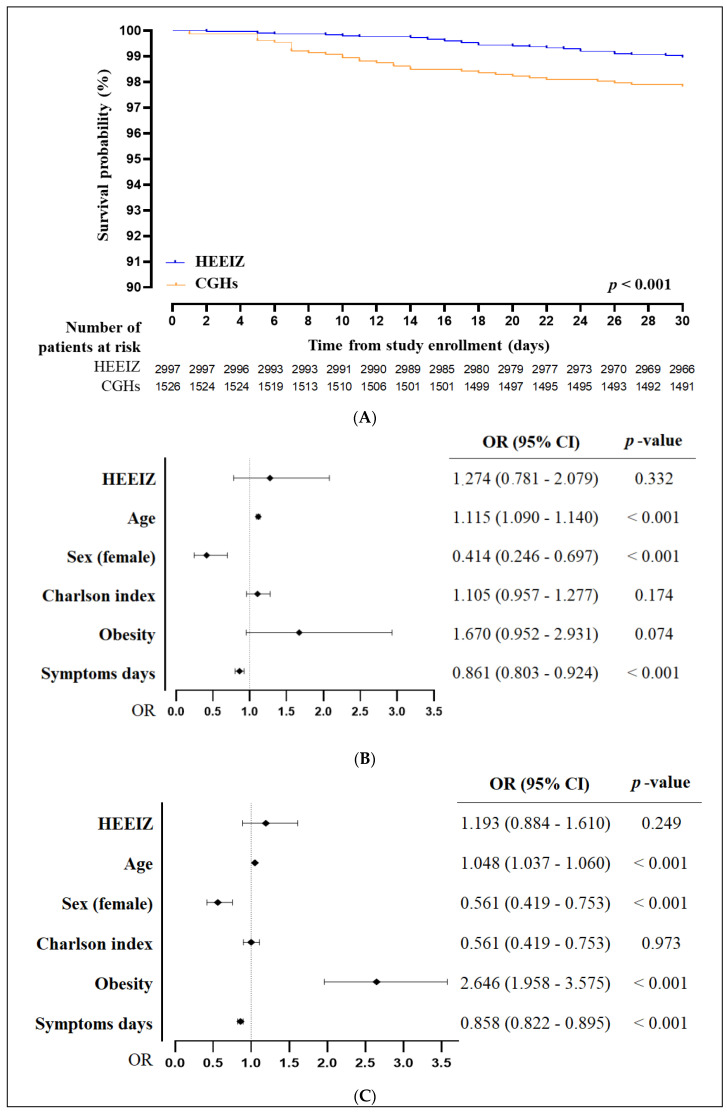
Course of primary objectives of mortality and IMV–death rate. (**A**) Cumulative incidence of 30-day survival according to curves from HEEIZ (blue) and CGHs (orange). (**B**) Multivariate regression analysis of mortality. (**C**) Multivariate regression analysis of IMV–death. Abbreviations—CGH: conventional general hospital; CI: confidence interval; HEEIZ: Hospital de Emergencias Enfermera Isabel Zendal; OR: odds ratio.

The 30-day survival analysis was significantly more favorable in the HEEIZ compared to CGHs (*p* < 0.001, Figure 2A); however, the adjusted HEEIZ hospital mortality was not significantly higher, compared with that of the CGHs (OR: 1.274; 95% CI: 0.781–2.079; *p* = 0.332). The variables that contributed significantly to the differences observed in mortality between the two cohorts are shown in Figure 2B,C. Older age contributed significantly to worse mortality and IMV–death rates (*p* < 0.001). Obesity was also associated with a worse IMV–death outcome (*p* < 0.001). Female sex and fewer symptomatic days at admission were both significantly protective for mortality and IMV–death (*p* < 0.001). Charlson index and HEEIZ hospitalization had no significant effects upon mortality and IMV–death ratios.

## 4. Discussion

This study showed that COVID-19 care at the HEEIZ dedicated center did not result in worse patient prognosis compared to that seen at CGHs, and was even associated with a trend towards improvement in several outcomes. Although previous studies have shown the benefits of dedicated centers in other countries in alleviating health challenges imposed by the SARS-CoV-2 pandemic [23,24,25,26], our study is the first to compare baseline characteristics, clinical management, treatments given, and outcomes of COVID-19 patients in a dedicated center vs CGHs during a challenging period of the pandemic in the Autonomous Region of Madrid (Spain).

We found that COVID-19 patients admitted to the HEEIZ had an overall better clinical outcome, lower mortality, and better IMV–death rates than those managed in CGHs. However, our multivariate regression analysis suggests that changes in these parameters are mainly attributable to the differences in baseline characteristics between the two cohorts. The variable of HEEIZ hospitalization alone did not significantly influence the clinical outcome in terms of mortality and IMV–death, either positively or negatively.

The study cohorts showed significant differences in several sociodemographic and clinical variables at baseline, with an overall lower age and a lower degree of comorbidities (Charlson index) among HEEIZ patients. Patients admitted to the CGHs were seven years older (median) than those admitted to the HEEIZ. Comorbidities have a direct and strong relation with older age, and furthermore, older age and the presence of comorbidities at admission are risk factors for COVID-19-related complications [27,28], explaining the lower baseline Charlson index observed in patients at the HEEIZ compared to CGHs. The treatment strategy of patients with COVID-19 pneumonia in the HEEIZ involved a more restricted use of glucocorticoids, remdesivir, antibiotics, and tozilizumab. In contrast, more oxygen therapy and noninvasive mechanical ventilation were used, with no difference in IMV. The clinical management differences observed could be explained by the specific characteristics of each COVID-19 protocol used and the availability of RICUs and ICUs. 

During the peaks of the COVID-19 pandemic, the HEEIZ accommodated up to 35% of the admissions related to this disease. This alternative facility setting was able to alleviate the burden on CGHs, enabling them to manage not only COVID-19 patients, but also other serious conditions requiring immediate attention. In fact, high ICU occupancy has been associated with increased mortality during the pandemic period [29]. Moreover, findings from RAND Corporation research have stressed that it is important for health resources to be allocated strategically during crises, not only to prevent system overload but also to stop maldistribution as to equitable care, supporting evidence-based first response and resilience [30]. Overall, these findings suggest that alternative health care institutions such as the HEEIZ not only supported the primary needs of COVID-19 patients, but also assisted the sustainability of regular hospitals, ensuring that complete healthcare services were provided during the unanticipated crisis. 

Based on WHO and ECDC international recommendations, joint monitoring systems have been implemented since late 2020 for influenza, SARS-CoV-2, and any other viruses capable of causing acute respiratory infection (ARI) in Spain [31]. The new ARI surveillance system (SiVIRA) has established sentinel surveillance in primary care and hospitals, with monitoring of seasonal epidemics of influenza, COVID-19, and respiratory syncytial virus. One of the key functions of the SiVIRA is to estimate the disease burden of respiratory viruses in Spain in order to guide decision-making and the planning of public health interventions when needed [32,33]. The SiVIRA also monitors outbreaks in situations that may require special public health actions to avoid hospitalizations and deaths [33,34]. In this respect, the role of specialized hospitals such as the HEEIZ could be crucial in future ARI health crises in Spain. The clinical approach discussed in this study could be easily standardized for other respiratory viruses, since they share several infective and prognostic risk factors. The HEEIZ has a capacity of 1000 beds, including 130 high-healthcare-demand beds (of which 96 and 34 are RICU and ICU beds, respectively) and an advanced ventilation system that allows air exchange 12 times an hour, helping to prevent viral spread.

One of the limitations of this study was that the decision on admission was left to the patient’s discretion, which resulted in a lack of stratification of baseline characteristics between the two cohorts. This influenced the clinical outcome, since the CGH cohort was older and presented more comorbidities at admission, resulting in worse survival and IMV–death ratios. Our electronic clinical history system also lacks certain registration capabilities, which restricted our clinical data collection. Another limitation was the relatively short recruitment period of 8 months. We decided to restrict our study to this period because it encompassed the most critical COVID-19 waves after the opening of the HEEIZ, prior to the improved epidemiologic control offered by the vaccination campaigns in the Autonomous Region of Madrid. In spite of this, our findings are valuable because they provide a clear perspective of the clinical outcomes obtained in each setting (dedicated hospital vs. CGH) with sufficient statistical power, due to the elevated number of patients included in each cohort. Since we provide robust evidence for the capacity of the HEEIZ to efficiently treat a constant number of COVID-19 patients, a capacity which progressively reduced the burden on CGHs during 2021, future studies could examine the economic impact that this dedicated hospital had in terms of administration and medical costs.

## 5. Conclusions

In conclusion, the HEEIZ increased its number of hospital admissions over time and reached up to 35% of new COVID-19 admissions in the entire Autonomous Region of Madrid. Despite the heterogeneity between clinical management protocols of COVID-19 patients with pneumonia among different hospitals, the clinical outcomes of patients treated at the HEEIZ were not significantly different compared to those admitted to CGHs. The HEEIZ helped free up beds in CGHs, allowing these hospitals to focus on specialized care and surgical activity for patients with other diseases. Follow-up studies should seek to determine the long-term clinical outcomes and the economic impact of dedicated COVID-19 treatment centers such as the HEEIZ. The lessons learned from the functioning of the HEEIZ would offer valuable insights into the planning and management of any future public health crisis, ensuring better responses within healthcare systems.

## Figures and Tables

**Table 1:** Patient demographic characteristics, comorbidities, and clinical/biochemical parameters at admission.

	Total Population (N = 4523)		HEEIZ Dedicated Center (N = 2997)	CGHs (N = 1526)	*p*-Value
	Valid *n*	Value			
Sex, male *n* (%)	4523	2642 (58.4)	1761 (58.8)	881 (57.7)	0.508
Age, median [25–75% IQR]	4523	54 (44–63)	52 (41–61)	59 (49–68)	<0.001
**Comorbidities**					
Charlson index, mean ± SD	4523	0.6 ± 1.2	0.5 ± 1	1 ± 1.5	<0.001
COPD, *n* (%)	4521	459 (10.2)	327 (10.9)	132 (8.7)	0.018
Obesity, *n* (%)	4521	750 (16.7)	444 (14.8)	306 (20.3)	<0.001
Days with symptoms, median (25–75% IQR])	4233	8 (6–10)	8 (6–10)	7 (5–10)	<0.001
Clinical parameters, median (25–75% IQR) *					
Systolic BP (mmHg)	4505	125 (114–140)	124.0 (113–137)	129.5 (118–144)	<0.001
Diastolic BP (mmHg)	4505	77 (70–84)	76 (70–84)	77 (70–85)	0.042
Heart rate (BPM)	4380	90 (79–101)	89 (78–100)	92 (81–103)	<0.001
Baseline oxygen saturation (%)	4515	95 (93–97)	95 (93–97)	95 (92–97)	<0.001
Respiratory rate (BPM)	3928	18 (16–22)	18 (16–21)	20 (15–24)	0.191
Temperature (°C)	4484	36.7 (36.1–37.3)	36.7 (36.2–37.5)	36.7 (36.2–37.3)	0.196
Laboratory variables, median [25–75% IQR] *					
CRP (mg/dL)	1724	28.6 (8.7–76.6)	35.7 (11.2–82.2)	15.3 (5.7–61)	<0.001
GOT (UI/L)	1501	38 (22–51)	40 (29–61)	36.5 (28–50)	<0.001
GPT (UI/L)	1631	37 (24–60)	39 (25–64)	33 (22.9–52)	<0.001
D-dimer (mg/L)	1560	487 (319.3–759.6)	459 (300–701)	558 (364–861)	<0.001
Creatinine (mg/dL)	1722	0.8 (0.6–1)	0.8 (0.7–1)	0.9 (0.7–1)	0.041
Sodium (mEq/L)	1712	137 (135–139)	137.5 (135–140)	137 (135–139)	0.016
Ferritin (µg/L)	869	552 (275–1053)	566 (269–1029)	543 (284–1075)	0.549
LDH (UI/L)	1523	317 (260–410)	308 (256–393)	336 (270–440)	<0.001
Leucocytes (×10^3^/µL)	1734	5960 (4600–7530)	5880 (4580–7400)	6200 (4840–8000)	0.004
Lymphocytes (×10^3^/µL)	1704	1000 (800–1400)	1060 (800–1400)	1000 (700–1680)	<0.001

* These parameters were registered in hospital emergency rooms (CGHs) on admission or day 1 (HEEIZ). BP: blood pressure; BPM: beats/breaths per minute; CGH: conventional general hospital; COPD: chronic obstructive pulmonary disease; CRP: C-reactive protein; GPT: glutamyl pyruvic transaminase; GOT: glutamyl oxaloacetic transaminase; HEEIZ: Hospital de Emergencias Enfermera Isabel Zendal; IQR: interquartile range (25–75%); LDH: lactate dehydrogenase; SD: standard deviation.

**Table 2:** Patient clinical progress during hospitalization.

	Total Population (N = 4523)		HEEIZ Dedicated Center (N = 2997)	CGHs (N = 1526)	*p*-Value
	Valid *n*	Value			
**Variable**					
**Length of hospital stay, days, median [25–75% IQR]**	4523	6 (4–10)	6 (4–10)	7 (5–11)	<0.001
**Destination a hospital discharge, *n* (%)**	4521				
Home		4108 (90.9)	2815 (93.9)	1293 (84.8)	<0.001
Social health center		180 (4.0)	11 (0.4)	169 (11.1)	
Hospital transfer		233 (5.2)	171 (5.7)	62 (4.1)	
Clinical complications, *n* (%) *	4523				
Respiratory failure		3618 (80)	2326 (77.6)	1292 (84.7)	<0.001
Sepsis		64 (1.4)	22 (0.7)	44 (2.9)	<0.001
Heart failure		59 (1.3)	33 (1.1)	26 (1.7)	0.091
Acute coronary syndrome		16 (0.4)	9 (0.3)	7 (0.5)	0.397
Renal failure		159 (3.5)	74 (2.5)	85 (5.6)	<0.001
Clinically relevant bleeding		56 (1.2)	36 (1.2)	20 (1.3)	0.752
Thromboembolic event		107 (2.4)	63 (2.1)	44 (2.9)	0.103
**Referrals (<60 days), *n* (%)**	4519	748 (16.7)	330 (11)	418 (27.4)	<0.001
COVID-19-related referrals, *n* (%)	748	246 (4.4)	146 (4.9)	100 (6.6)	0.018
Days until referral, median [25–75% IQR]	748	18 (7–32)	17 (7–32)	19 (8–32)	0.343
Admission after referral, *n* (%)	748	87 (11.8)	35 (10.6)	52 (12.7)	0.383
**IMV, *n* (%)**	4523	222 (4.91)	134 (4.5)	88 (5.8)	0.057
**Hospital mortality, *n* (%)**	4523	97 (2.1)	46 (1.5)	51 (3.3)	<0.001
Days until death, median [25–75% IQR]	97	21 (11–34)	24 (16–32)	18 (8–36)	0.298
**IMV–death, *n* (%)**	4523	263 (5.8)	147 (4.9)	116 (7.6)	<0.001

CGH: conventional general hospital; HEEIZ: Hospital de Emergencias Enfermera Isabel Zendal; IMV: invasive mechanical ventilation; IQR: interquartile range (25–75%). * Criteria and thresholds are described in Supplementary Table S1.

## Data Availability

The data that support the findings of this study are available on request from the corresponding author. These data are not publicly available due to privacy or ethical restrictions.

## Supplementary Materials

The following supporting information can be downloaded at: https://www.mdpi.com/article/10.3390/healthcare12141365/s1. Table S1: Criteria and thresholds used to define clinical complications described in Table 2; Figure S1: HEEIZ occupation from 20 December 2020 to 31 December 2021. (A) Total daily HEEIZ occupancy during the three COVID-19 waves in this year. (B) Number of COVID-19 patients newly admitted to the HEEIZ and in the entire Autonomous Region of Madrid during 2021 [22], and as relative percentages. HEEIZ: Hospital de Emergencias Enfermera Isabel Zendal; Figure S2: Patient screening and inclusion flow chart. CGH: conventional general hospital; HEEIZ: Hospital de Emergencias Enfermera Isabel Zendal; List of additional contributors.
