# Supplementary material for: Effectiveness of Treatment Approaches in COVID-19 Pneumonia: A Comparative Evaluation between a Specialized Center and Conventional Hospitals
Source: Healthcare (Basel). 2024 Jul 9;12(14):1365. doi: 10.3390/healthcare12141365 (PMC11276510; doi:10.3390/healthcare12141365)
Supplement: Supplementary file 1 [file healthcare-12-01365-s001.zip › healthcare-3048354-supplementary.pdf]

SUPPLEMENTARY MATERIAL

Effectiveness of treatment approaches in COVID-19 pneumonia: a comparative evaluation between a specialized center and conventional hospitals

Supp. Table 1. Criteria and thresholds used to define clinical complications described in Table 2

**Clinical complication**	**Criteria and threshold**
Respiratory failure	Pulse oximetry: <92% saturation<br>or<br>PaO2: <60 mmHg
Sepsis	qSOFA score ≥ 2
Heart failure	Compatible parameters<br>and<br>NT-proBNP >1800<br>and<br>Lung congestion determined by X-ray or US
Acute coronary syndrome	Clinical signs and ECG changes with elevation of markers of myocardial damage
Renal failure	As specified in patient clinical history
Clinically relevant bleeding	Blood transfusion needed
Thromboembolic event	Evidence of venous thrombosis in the extremities or lung, diagnosed by US or CT scan, respectively.

CT: computed tomography; ECG: electrocardiogram; NT-proBNP: N-terminal prohormone of brain natriuretic peptide; PaO2: partial pressure of oxygen; qSOFA: quick Sequential Organ Failure Assessment; US: ultrasound.

Supp. Figure 1. HEEIZ occupation from 20 December 2020 to 31 December 2021. (A) Total daily HEEIZ occupancy during the three COVID-19 waves in this year. (B) Number of COVID-19 patients newly admitted to the HEEIZ and in the entire Autonomous Region of Madrid during 2021 [22], and as relative percentage.

A

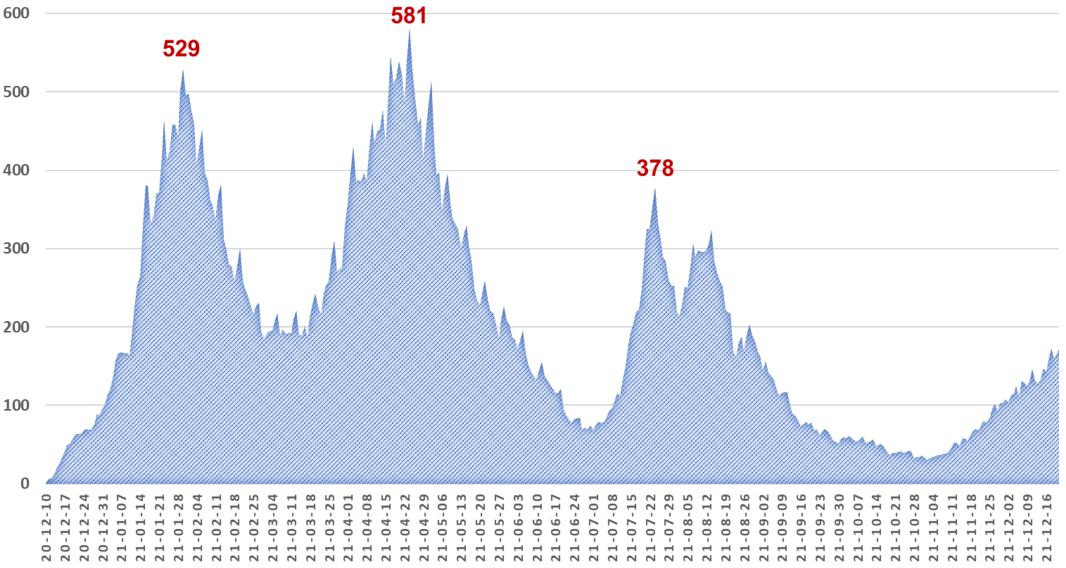

B

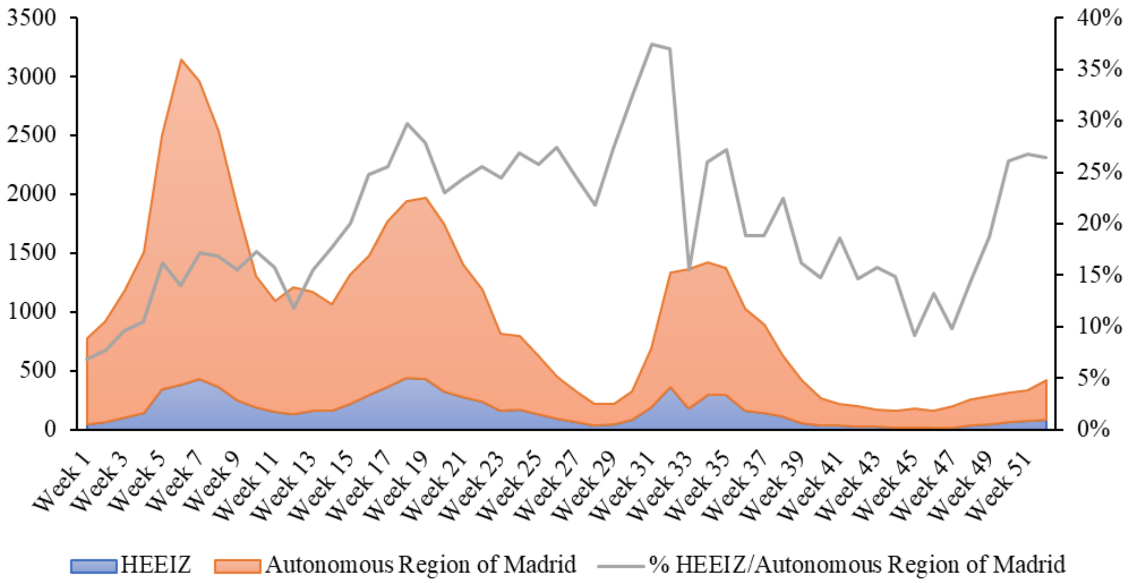

HEEIZ: Hospital de Emergencias Enfermera Isabel Zendal.

Supp. Figure 2. Patient screening and inclusion flow chart.

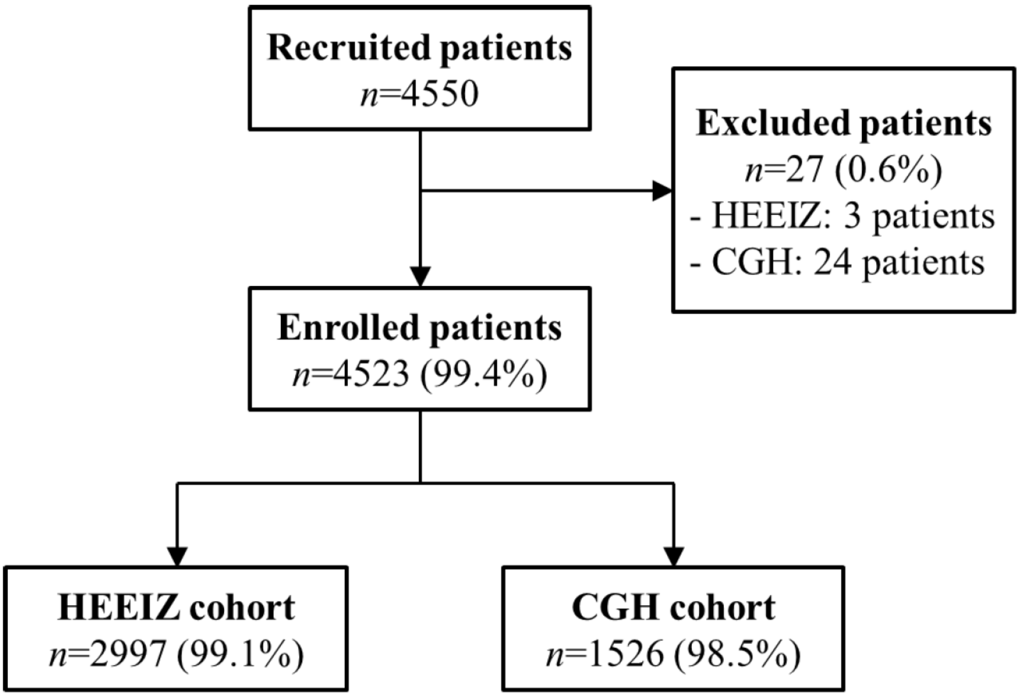

CGH: conventional general hospital; HEEIZ: Hospital de Emergencias Enfermera Isabel Zendal.

LIST OF ADDITIONAL CONTRIBUTORS

Hospital del Henares

Cristóbal Rodríguez Leal, Raquel Barrós González, Aranzazu Galindo Martín, Javier Díaz Luperena, Laura Mao Martín, Ana Chica Benayas, Eloisa Delgado Torres, Manuel Medina Pedrique, Ana Osuna Baillo.

Hospital Universitario 12 de Octubre

Antonio Blanco Portillo, Laura Álvarez Santiago, Noelia García Barrio.

Hospital Universitario Puerta de Hierro-Majadahonda

Giselle Santos Rielo, Mónica Enriquez botas, Estefanía de las Heras Bahos.

Hospital Clínico Universitario San Carlos

Juan Gonzalez del Castillo, Carlota Clemente Callejo, Eric Jorge García-Lamberechts, Sara Lainez Martínez.

Hospital Universitario Severo Ochoa

Irene Cabrera Rodrigo, Verónica Mercado Valdivia, Teresa Agudo Villa.

Hospital Universitario Getafe

María Teresa del Cerro Saelices, Miguel Muñoz Flores, Corina Cazorla Cana, Ana Herrera Rodríguez, Mariella Luengo López, Silvia Odeh Santana, Cecilia Granda París, Juan Pedro Zabaleta Camino, Manuel Jesús Ruiz Polaina.

Hospital General Universitario Gregorio Marañón

Esther Gargallo Garcia, Jose Antonio Sevillano Fernando, Juan Antonio Andueza Lillo.

Hospital Universitario La Princesa

Guillermo Fernández, Carmen del Arco Galán.

Hospital Universitario La Paz

María Angélica Rivera Nuñez, Susana Martínez Álvarez, Elena Calvin García, Ana Martínez Virto, Ángeles Molina Medina, Begoña Reche Martínez, Charbel Maroun Eid, Elena Muñoz del Val, Macarena Lerín Baratas, Nataly Cancelliere Fernández.

Hospital del Sureste

Marta López Chousa, Alejandro Valencia Dutor.

Hospital Universitario Infanta Leonor

José Costell Jaime, María Mir Montero, Carlos Bibiano Guillén.

Hospital Emergencias Enfermera Isabel Zendal

Jorge Carriel Mancilla, Marcos Fragiel-Saavedra, Pedro Landete Rodríguez, María de Lara Gutierrez García, Antonio Jimenez Moreno, Concepción Martinez-fidalgo Vázquez, Eva Prats García, Eduardo Cano Sanz, María Ángeles Ortega Fraile, José Terán Tiñedo, Tomás Villén Villegas, Ángela Trueba Vicente, Ignacio Puyol Varela.
